# Supplementary material for: Analysis of PPARγ Signaling Activity in Psoriasis
Source: Int J Mol Sci. 2021 Aug 10;22(16):8603. doi: 10.3390/ijms22168603 (PMC8395241; doi:10.3390/ijms22168603)
Supplement: Supplementary file 1 [file ijms-22-08603-s001.zip › Supplemental materials_Analysis of PPARg signaling activity in psoriasis/Pathway models/Models images and html files/Model of PPARG signaling in psoriasis/6581.html]

ITPR1


# Protein ITPR1

|  |  |
| --- | --- |
| URN | urn:agi-llid:3708 |
| Total Entities | 0 |
| Connectivity | 1313 |
| Name | ITPR1 |
| Description | inositol 1,4,5-trisphosphate receptor, type 1 |
| Notes | This gene encodes an intracellular receptor for inositol 1,4,5-trisphosphate. Upon stimulation by inositol 1,4,5-trisphosphate, this receptor mediates calcium release from the endoplasmic reticulum. Mutations in this gene cause spinocerebellar ataxia type 15, a disease associated with an heterogeneous group of cerebellar disorders. Multiple transcript variants have been identified for this gene. [provided by RefSeq, Nov 2009] |
| Primary Cell Localization | Cytoplasm |
| Class | Transporter |
| ObjectType | Protein |

---

|  |  |
| --- | --- |
| Pathway | Alzheimer's Disease |
|  | Circadian Clock Genes Mutations Cause Insomnia |
|  | Skin Aging |
|  | High Fat Diet Activates cAMP Related Exocytosis in Synapse and Endocrine Cell |
|  | GCG and PPY Regulate Metabolism and Satiety |
|  | mTOR Signaling Activation by Taste Sensor Receptors (Rodent Model) |
|  | Taste Receptors Activation in Tongue |
|  | GUCY2C Signaling in Apetite Regulation |
|  | L-cell: GCG, PYY and 5-HT Release |
|  | P-cell Activation |
|  | I-cell: CCK-8 Secretion and Regulation of Eating Behavior |
|  | D-cell: Somatostatin Release |
|  | A-cell (X-like): Ghrelin and Nesfatin-1 Synthesis |
|  | G-cell: Gastrin Secretion |
|  | ECL-cell: CHGA and Histamine Synthesis |
|  | Insulin Secretion |
|  | Dopamine Receptors Signaling |
|  | Serotonin Receptors Signaling |
|  | Catecholamines Secretion from Adrenal Gland |
|  | Thyroid Hormones Common non-Genomic Effects in Hyperthyroidism |
|  | Circadian Clock and Sleep Regulation by Melatonin |
|  | Norepinephrine Release Regulation |
|  | Dopamine Synthesis and Release |
|  | CRH Secretion Regulation |
|  | Prolactin Production |
|  | Oxytocin Action in Mammary Gland |
|  | Thrombopoietin Receptors Signaling in Platelet Maturation |
|  | Mineralcorticoids Secretion Regulation |
|  | Dectin-1 (CLEC7A) Signaling |
|  | Dectin-2 (CLEC6A)/Mincle (CLEC4E)/BDCA2 (CLEC4C) Signaling |
|  | T-Cell Dependent B-Cell Activation |
|  | T-Cell Independent B-Cell Activation |
|  | V(D)J Recombination Activation |
|  | Natural Killer Cell Activation through C-Type Lectin-like Receptors |
|  | Natural Killer Cell Activation through ITAM-Containing Receptors |
|  | Natural Killer Cell Activation through ITSM-Containing Receptors |
|  | T-Cell Maturation (Hypothesis) |
|  | Natural Killer Cell Activation |
|  | T-Cell Central Tolerance |
|  | CD8+ T-Cell Activation |
|  | Th1-Cell Differentiation |
|  | Treg-Cell Differentiation |
|  | Th2-Cell Differentiation |
|  | T-Cell Receptor Signaling |
|  | CXC Chemokine Receptor Signaling |
|  | CC Chemokine Receptor Signaling |
|  | Platelet Activation via Adhesion Molecules |
|  | Scavenger Receptors in Platelet Activation |
|  | Scavenger Receptors in Platelet Aggregation |
|  | Proplatelet Maturation |
|  | Bradykinin Effects in Inflammation |
|  | Platelet Activation via GPCR Signaling |
|  | Lipoxin A4/FPR2-Related Neutrophil Depression |
|  | Annexins in Inflammation Resolution |
|  | Eat me Signal: Apoptotic Cell Induces Phagocytosis |
|  | Leukotriene Effect on Vascular Endothelial Cell Response |
|  | Eicosanoids in Myeloid Cell Activation |
|  | Leukotriene Effect on Airway Smooth Muscle Response |
|  | Histamine Receptor H3 Signaling in Neurons |
|  | Prostaglandin E2 and Histamine in Gastric Acid Secretion |
|  | Basophil Activation |
|  | Eosinophil Activation |
|  | Mast-Cell Activation via IgE Signaling |
|  | Mast-Cells De Novo Synthesized Mediators via IgE Independent Signaling |
|  | CD16/CD14 Proinflammatory Monocyte Activation |
|  | Macrophage M2-Related Phagocytosis |
|  | anti-Inflammatory Function of Macrophage M2 Lineage |
|  | Neutrophil Recruitment and Priming |
|  | Neutrophil Activation via FCGR3B |
|  | Neutrophil Degranulation via FPR1 Signaling |
|  | CR3 Mediated Phagocytosis in Neutrophils and Macrophages |
|  | Neutrophil Chemotaxis |
|  | Neutrophil Activation via Adherence on Endothelial Cells |
|  | SNS Related Adrenal Cortex Response |
|  | Vascular Smooth Muscle Cell/Pericyte Migration and Proliferation |
|  | Vascular Endothelial Cell Activation by Cytokines |
|  | Vascular Endothelial Cell Activation by Blood Coagulation Factors |
|  | Vascular Endothelial Cell Permeability Activation |
|  | Potassium Flux Regulation |
|  | GFs/TNF -> Ion Channels |
|  | Nociception Expression Targets Signaling |
|  | Vascular Motility |
|  | Ca2+ Flux Regulation |
|  | TAAR1 -> Neurotransmitter Uptake |
|  | NPY1R -> CRH/POMC Production |
|  | NTSR1 Expression Targets |
|  | CRH -> Synthesis of Corticosteroids |
|  | EDNRA/B -> Vascular Motility |
|  | CCKBR -> Neurotransmitter Uptake |
|  | ADORA3 -> Mast-Cell Degranulation |
|  | ADRA1 -> Vasoconstriction |
|  | ADRA1A -> IL6 Production |
|  | ADRA1 -> Ion Channels |
|  | ADRA2C/ADRB2 -> Vasoconstriction |
|  | ADRA2C/ADRB2 -> Synaptic Endocytosis |
|  | ADRA1 -> Prostaglandin Generation |
|  | BDKRB1/2 -> Ion Channels |
|  | BDKRB1/2 -> Prostaglandin Generation |
|  | CNR1/2 -> Membrane Transport |
|  | CNR1/2 -> Vascular Motility |
|  | CNR1/2 -> IL1B/2/4/6/10 Production |
|  | DRD2 Expression Targets |
|  | GALR1/2/3 -> POMC/NPY Production |
|  | GALR1/2/3 -> Neurotransmitter Metabolism |
|  | HRH1/2 -> Membrane Polarization |
|  | HRH1/3 -> Synaptic Transmission |
|  | HRH1/2 -> Vascular Motility |
|  | HRH2/4 -> IL6/10 Production |
|  | CHRM1 -> IL2 Production |
|  | CHRM1/2/3/5 -> Ion Channels |
|  | CHRM1/2/3 -> Vascular Motility |
|  | OPRK -> Pain Perception |
|  | PTGFR -> Vasoconstriction |
|  | PTGIR -> IL6 Production |
|  | PTGER1/4 -> Vascular Motility |
|  | PTGER2/3 -> Vascular Motility |
|  | PTGER2/3 -> Inflammation-Related Expression Targets |
|  | HTR1 -> IL6 Production |
|  | HTR5 -> TNF Production |
|  | HTR2 -> Membrane Transport |
|  | HTR1 -> Membrane Transport |
|  | HTR1 -> Vascular Motility |
|  | TACR1 -> Membrane Transport |
|  | TACR1 -> TNF/IL6/IL8 Production |
|  | GRM1/5 (Postsynaptic) -> Ion Channels |
|  | P2RY11/13/14 -> IL8/10 Production |
|  | P2RY1/2/4/6 -> Potassium Channels |
|  | NTRK1/2/3 -> Acetylcholine Production |
|  | EPHB -> NMDA Receptor Activation |
|  | Luteinizing Hormone in Oocyte Meiotic Resumption |
|  | Agomelatine Antidepressant Action |
|  | Histamine in Arousal Regulation |
|  | Circadian Clock in Sleep Regulation |
|  | Endocannabinoids Role in Sleep Regulation |
|  | Circadian Clock Genes in Suprachiasmatic Nuclei Neurons |
|  | Hypocretin (Orexin) in Arousal Regulation |
|  | Adenosine Role in Sleep Regulation |
|  | Retinal Ganglion and Suprachiasmatic Nucleus in Melatonin Synthesis Regulation |
|  | G0/G1 Phase Transition |
|  | Gap Junction Assembly |
|  | TRRAP/TIP60 in Chromatin Remodeling |
|  | Th1-Cells Activation and Proliferation in Atherosclerosis |
|  | Proteins Involved in Arterial Hypertension |
|  | Endothelin Signaling in Arterial Hypertension |
|  | Arterial Hypertension |
|  | Aldosterone Synthesis Acute Regulation |
|  | Endothelial Cell Dysfunction in Arterial Hypertension |
|  | Aldosterone Synthesis Chronic Regulation |
|  | Smooth Muscle Cell Dysfunction in Arterial Hypertension |
|  | TRPM4/6/7/8 Signaling in Arterial Hypertension (Hypothesis) |
|  | Smooth Muscle Cell Dysfunction in Pulmonary Hypertension |
|  | Myocardial Remodeling |
|  | Vascular Reactivity in Raynaud Disease |
|  | Cold Stress and Adrenoceptor alpha 2C Signaling in Raynaud Disease |
|  | NO Synthesis in Early Vasospasm |
|  | Contraction Due Vasospasm |
|  | Atopic Dermatitis Onset |
|  | Mast-Cells Activation in Atopic Dermatitis |
|  | Cell Cycle Dysregulation in Melanoma |
|  | WNT Signaling in Melanoma |
|  | Melanoma |
|  | Apoptosis Block in Melanoma Cell |
|  | MITF as a Regulator of Melanoma Cell Development |
|  | Th0-Cell Aberrant Activation in Systemic Lupus Erythematosus |
|  | Th1-Cell Function in Systemic Lupus Erythematosus |
|  | B-Cell Activation in Systemic Lupus Erythematosus |
|  | Th2-Cell Function in Systemic Lupus Erythematosus |
|  | Acute Cytotoxic CD8+T-Cell Response against Melanocytes |
|  | Vitiligo |
|  | T-Cell Cytotoxic Response against Melanocytes in Vitiligo |
|  | Apoptosis of Melanocyte in Vitiligo |
|  | Humoral Immunity in Vitiligo |
|  | CD8+ T-Cell Response to Self-Determinants in Diabetes |
|  | Treg-Cell Function in Diabetes Mellitus Type 1 |
|  | Natural Killer T-Cell Roles in Diabetes Mellitus Type 1 |
|  | non-Suppressive Treg-Cell in Diabetes Mellitus Type 1 |
|  | TLR2 Signaling in Treg-Cell in Type 1 Diabetes (Animal Model) |
|  | Natural Killer Cell in Diabetes Mellitus Type 1 |
|  | Peripheral Tolerance to Autoantigens Recession in Diabetes Mellitus Type 1 |
|  | Treg-Cell Activation in Diabetes Mellitus |
|  | Proliferative Diabetic Retinopathy |
|  | G-Proteins Signaling in Insulin Secretion in beta-Cell |
|  | Olanzapine Effect on Insulin Secretion in Diabetes Mellitus |
|  | Bone Remodeling in Hyperthyroidism |
|  | Cardiovascular Effects in Hyperthyroidism |
|  | Thyroid Dysfunction |
|  | Immune System Activation in Hyperthyroidism |
|  | GNRH Release Impairment in Hypogonadotrophic Hypogonadism |
|  | GNRH Signaling Impairment in Hypogonadotrophic Hypogonadism |
|  | KISS1 Production Impairment in Hypogonadotropic hypogonadism |
|  | Hypothyroidism, Secondary (Central) |
|  | Thyroid Stimulating Hormone (TSH) Resistance in Congenital Hypothyroidism |
|  | Overt Hypothyroidism, Primary |
|  | Thyroid Stimulating Hormone (TSH) Secretion in Overt Hypothyroidism |
|  | Central Hypothyroidism |
|  | Tertiary Hypothyroidism |
|  | Prolactinoma Overiew |
|  | CD4+T-Cell Response in Celiac Disease |
|  | CD8+ T-Cell Response in Celiac Disease |
|  | Metastatic Colorectal Cancer |
|  | B-Cell Activation in Crohn's Disease |
|  | OipA Signaling in Helicobacter Pylori Infection |
|  | CagA Phosphorylation Dependent Signaling |
|  | EDN, NRG, NRTN, and GDNF/RET Signaling in Hirschsprung Disease |
|  | Th2-Cell Function in Ulcerative Colitis |
|  | T-Cell Cytotoxic Response in Ulcerative Colitis |
|  | Antiphospholipid Antibodies in Platelets and Coagulation Cascade |
|  | B-Cell Chronic Lymphocytic Leukemia |
|  | Diffuse Large-B-Cell Lymphoma |
|  | Diffuse Large-B-Cell Lymphoma ABC Subtype |
|  | Hodgkin Lymphoma |
|  | Mantle Cell Lymphoma |
|  | mTOR/NF-kB/BCR Signaling Disregulation in Mantle Cell Lymphoma |
|  | Mucosa-Associated Lymphoid Tissue (MALT) Lymphoma |
|  | Splenic Marginal Zone Lymphoma |
|  | Platelet and Coagulation Cascade Activation by Antiphospholipid Antibodies |
|  | T-Cell Acute Lymphoblastic Leukemia |
|  | TRPM7/8 in Pancreatic Adenocarcinoma |
|  | Eosinophil Survival in Asthma |
|  | Eosinophil Activation and Degranulation in Asthma |
|  | Airway Smooth Muscle Cell High Rate Proliferation |
|  | IgE Induces Airway Smooth Muscle Cell Proliferation |
|  | Airway Smooth Muscle Cell Contraction |
|  | Goblet-Cell Exocytosis in Asthma |
|  | Neutrophil Degranulation via FPR1/IL8 |
|  | Basophil Activation in Asthma |
|  | Mast-Cell Activation in Asthma |
|  | Mast-Cell Related Chemokines Signaling in Asthma |
|  | Antigen-Presenting Cell Role in Asthma |
|  | Th2-Cell Response in Asthma |
|  | CFTR Facilitates Oxidative Stress in Airway Epithelium in CF |
|  | Airway Epithelial Cell Dysfunction |
|  | CFTR Expression in Epithelial Cells (Class I Mutations) |
|  | CFTR Related Ion-Channel Dysfunction in Cystic Fibrosis Airway Epithelium (Class III Mutations) |
|  | Steroidogenesis Impairement in Polycystic Ovary Syndrome |
|  | Ovulation Block |
|  | Polycystic Ovary Syndrome |
|  | FSH Action in Polycystic Ovary Syndrome |
|  | PTPN22 Role in Rheumatoid Arthritis |
|  | B-Cells Function in Systemic Scleroderma |
|  | Th2-Cells Function in Systemic Scleroderma |
|  | WHIM Syndrome |
|  | Dentin Formation Disruption |
|  | CD8+ T-Cell Action Impairment in HIV Type 1 Infection |
|  | CD4+ T-Cell Function Decline in HIV |
|  | Acinar Cells in the Saliva Formation |
|  | Epithelial Cell in the Saliva Formation |
|  | Proteins Involved in Friedreich Ataxia |
|  | Glutamate Toxiicity Effects on Striatum Medium Spiny Neurons in Hantington's Disease |
|  | ER Stress in Huntington Disease |
|  | Proteins Involved in Huntington Disease |
|  | Ca2+ Toxicity in Neurons |
|  | BDNF Upregulation Triggered by Seizures |
|  | Blood-Brain Barrier Disruption in Epileptiform Disorders |
|  | mTOR Hyperactivation after Status Epilepticus |
|  | Glutamate, D-Serine, and ATP Release from Astrocytes |
|  | Glioblastoma, Primary |
|  | Glioblastoma, Secondary |
|  | Astrocytoma |
|  | Vascular Endothelial Cell Vasodilation in Brain |
|  | CCL2 Role in CGRP Expression and Release |
|  | Dopamine Synthesis and Release in Migraine |
|  | Vascular Smooth Muscle Cell Vasodilation in Brain |
|  | Postsynaptic Neuron Activation |
|  | eNOS (NOS3) Activation via Prostaglandin E2 and Histamine |
|  | NO Effect on Vasodilation and Vasoconstriction |
|  | GABRB3 Mutation and Adenosine Deaminase Polymorphism |
|  | Hypocretin Elevation and Monoamine Oxidase Decrease/Inhibition |
|  | Angiogenesis in Endometriosis |
|  | Coagulation Factors and PAR1/2 Receptors (F2R/F2RL1) in Endometriosis |
|  | EGFR and PTAFR Signaling in Ovarian Cancer |
|  | HPV E5 Regulates EDNRA and EGFR Signaling Pathway in Keratinocytes |
|  | Prostate Cancer |
|  | TRPM8 Effects in Prostate Cancer (Hypothesis) |
|  | Oxidative Stress, All-Trans-Retinal and Lipofuscin Toxicity in AMD |
|  | Ca2+ Toxicity in Lens Cells |
|  | VEGFA in Neovascular Glaucoma |
|  | Glutamate Mediated Excitotoxicity in Glaucoma |
|  | PGF2-alpha Effect on Treatment |
|  | Serotonin Signaling in the Ciliary Body |
|  | Cytokines Trigger Otitis Media |
|  | Alveolar Macrophages Dysfunction in COPD |
|  | Muscarinic Receptor-Mediated Airway Smooth Muscle Contraction |
|  | Alveolar Epithelial Cell Dysfunction in COPD |
|  | Penile Detumescense Augmentation Mechanisms |
|  | Endothelial Cell Dysfunction in Glomerulonephritis |
|  | Sertoli Cells Dysfunction Causes Male Infertility |
|  | CD38/CD3 -> JUN/FOS/NF-kB Signaling in T-cell Proliferation |
|  | CD38/CD19 -> JUN/FOS/NF-kB Signaling in B-cell Proliferation |
|  | CD38 -> cADPR/Calcium Signaling |
|  | CD157 -> cADPR/Calcium Signaling |
|  | ANGPT1 -> CREB/CREBBP Expression Targets |
|  | ANGPT2 -> AP-1/CREBBP/MYC Expression Targets |
|  | AREG -> NFATC Expression Target |
|  | AREG -> CREB/CREBBP Expression Targets |
|  | AREG -> NCOR2 Expression Targets |
|  | AREG -> SMAD1 Expression Target |
|  | AREG -> HIF1A Expression Targets |
|  | AREG -> AP-1 Expression Targets |
|  | Noradrenaline/Gs Expression Targets |
|  | Noreadrenaline/Gq Expression Targets |
|  | Epinephrine/Gs Expression Targets |
|  | Acetylcholine Expression Targets |
|  | Serotonin/Gq Expression Targets |
|  | Epinephrine/Gq Expression Targets |
|  | Dopamine/Gi Expression Targets |
|  | B-Cell Receptor -> NFATC Signaling |
|  | B-Cell Receptor -> AP-1 Signaling |
|  | B-Cell Receptor -> NF-kB Signaling |
|  | BTC -> NFATC Expression Targets |
|  | BTC -> AP-1/ATF/CREB Expression Targets |
|  | BTC -> EP300/ETS/ETV/SP1 Expression Targets |
|  | SELE -> ELK-SRF Signaling |
|  | CD2 Expression Targets |
|  | CD19 Expression Targets |
|  | CD81 -> AP-1 Expression Targets |
|  | CD247 Expression Targets |
|  | gamma Globulins Expression Targets |
|  | FCGR3A Expression Targets |
|  | CD19/CR2 Expression Targets |
|  | CD8 -> AP-1 Expression Targets |
|  | CD72 -> CREB/CREBBP Expression Targets |
|  | CD72 -> NF-kB Expression Targets |
|  | CD8 -> ATF/CREB/CREBBP Expression Targets |
|  | CD8 -> NF-kB Expression Targets |
|  | CD80 -> AP-1 Expression Targets |
|  | CD81 Expression Targets |
|  | CD80 -> NF-kB Expression Targets |
|  | CD80 -> NFATC Expression Targets |
|  | CD86 -> NFATC Expression Targets |
|  | CD86-> NFATC Expression Targets |
|  | CD86 -> NF-kB Expression Targets |
|  | CXCL6 Expression Targets |
|  | CXCL1 Expression Targets |
|  | CXCL2 Expression Targets |
|  | CXCL3 Expression Targets |
|  | CXCL5 Expression Targets |
|  | Complement Component GPCRs Signaling |
|  | Clozapine Induced Granulocytopenia |
|  | EGF -> HIF1A Expression Targets |
|  | EGF -> AP-1/ATF Expression Targets |
|  | EGF -> CREB/CREBBP/ELK/SRF/MYC Expression Targets |
|  | EGF -> MEF/MYOD/NFATC Expression Targets |
|  | EGF -> TP53 Expression Targets |
|  | EGF -> NCOR2 Expression Target |
|  | EGFR -> NCOR2 Signaling |
|  | EGFR/ERBB3 -> MEF/MYOD/NFATC/MYOG Signaling |
|  | EGFR/ERBB2 -> TP53 Signaling |
|  | ERBB2/3 -> EP300/ETS/ETV/SP1 Signaling |
|  | EGFR -> SMAD1 Signaling |
|  | EGFR/ERBB2 -> HIF1A Signaling |
|  | EGFR -> AP-1/ATF2 Signaling |
|  | EGFR -> AP-1/CREB/ELK/SRF/MYC Signaling |
|  | EREG -> CREB Expression Target |
|  | EREG -> EP300/SP1 Expression Targets |
|  | EREG -> HIF1A Expression Target |
|  | EREG -> AP-1/ATF Expression Targets |
|  | Erythropoietin -> AP-1/MYC/CREB Expression Targets |
|  | Erythropoietin -> ELK/SRF Expression Targets |
|  | F2 -> AP-1/CREB/ELK/SRF/SP1 Expression Targets |
|  | PLG -> AP-1/CREB/ELK/SRF/SP1 Expression Targets |
|  | FGF9 -> AP-1/CREB/MYC Expression Targets |
|  | FGF10 -> AP-1/CREB/CREBBP/MYC Expression Targets |
|  | FGF1 -> AP-1/CREB/ELK/SRF/MYC Expression Targets |
|  | FGF1 -> NCOR2 Expression Target |
|  | FGF7 -> RUNX Expression Targets |
|  | FGF8 -> AP-1/CREB/MYC Expression Targets |
|  | FGF1 -> RUNX Expression Targets |
|  | FGF18 -> AP-1/CREB Expression Targets |
|  | FGF23 -> NCOR2 Expression Targets |
|  | FGF8 -> RUNX Expression Targets |
|  | FGF9 -> RUNX Expression Targets |
|  | FGF2 -> NCOR2 Expression Targets |
|  | FGF4 -> AP-1/MYC Expression Targets |
|  | FGF7 -> AP-1/CREB/CREBBP/MYC Expression Targets |
|  | FGF2 -> RUNX Expression Targets |
|  | FGF2 -> AP-1/CREB/CREBBP/ELK/SRF/MYC Expression Targets |
|  | FGFR -> RUNX2 Signaling |
|  | FGFR -> AP-1/CREB/CREBBP/ELK/SRF/MYC Signaling |
|  | FPR1 -> Cytoskeleton Signaling |
|  | ADCYAP1 Expression Targets |
|  | FSHR Expression Targets |
|  | Glutamate/Gq Expression Targets |
|  | VIP Expression Targets |
|  | HBEGF -> AP-1/ATF Expression Targets |
|  | HBEGF -> TP53 Expression Targets |
|  | HBEGF -> EP300/ETS/ETV/SP1 Expression Targets |
|  | HBEGF -> HIF1A Expression Targets |
|  | HBEGF -> MEF/MYOD Expression Target |
|  | HBEGF -> CREB/MYC Expression Targets |
|  | HGF -> AP-1/CREB/ELK/SRF/MYC Expression Targets |
|  | HGFR -> AP-1/CREB/MYC Signaling |
|  | EGFR Transactivation in Cancer and Non-Cancer Cells |
|  | TAM Receptor Signaling Activation in Cancer |
|  | G0/G1 Cell Cycle Phase Transition Activation in Cancer |
|  | NFKB Canonical Signaling Activation in Cancer |
|  | VEGF Independent Angiogenesis in Cancer |
|  | CDH2 Activation Promotes Cancer Cell Migration and Survival |
|  | Treg Cells Promote Immunosuppression in Cancer Immune Escape |
|  | CD2 -> NFATC1 Signaling |
|  | CD19 -> JUN/ELK Signaling |
|  | FcIgER -> NFATC1 Signaling |
|  | FcIgER -> ELK-SRF Signaling |
|  | ICAM1 -> AP-1/CREB/ELK/SRF Signaling |
|  | FibronectinR -> AP-1/ELK/SRF/SREBF Signaling |
|  | CTGF -> AP-1/CREB/MYC Expression Targets |
|  | CTGF -> NCOR2 Expression Target |
|  | ICAM1 Expression Targets |
|  | IL16 -> AP-1 Expression Targets |
|  | IL16-> ATF/CREB/CREBBP Expression Target |
|  | UrokinaseR -> ELK/SRF Signaling |
|  | PTPRC -> BCL6 Expression Targets |
|  | Prostaglandin F Expression Targets |
|  | Thromboxane A2 Expression Targets |
|  | LPA Expression Targets |
|  | PAF/Gq -> AP-1/ATF1/CREB/ERK/SRF Expression Targets |
|  | Dronabinol/Anandamide Expression Targets |
|  | Morphine Expression Targets |
|  | PAF/Gq -> NF-kB Expression Targets |
|  | S1P Expression Targets |
|  | PGE1 Expression Targets |
|  | NRG1 -> MEF/MYOD Expression Targets |
|  | NRG1 -> EP300/ETS/ETV/SP1 Expression Targets |
|  | NRG1 -> CREB/CREBBP/ELK/SRF/MYC Expression Targets |
|  | NRG1 -> TP53 Expression Target |
|  | NRG1 -> AP-1/ATF Expression Targets |
|  | NRG1 -> HIF1A Expression Target |
|  | NGF -> SMAD3/NF-kB Expression Targets |
|  | NGF -> AP-1/TP53/MYC Expression Targets |
|  | NGF -> FOXO/MYCN/ELK-SRF Expression Targets |
|  | BDNF Expression Targets |
|  | NTF3 Expression Targets |
|  | NTF4 Expression Targets |
|  | NGF -> CREB/CEBPB/MEF2A Expression Targets |
|  | Adenosine Expression Targets |
|  | PDGFB -> AP-1/CREB/MYC Expression Target |
|  | PDGFC -> CREB Expression Target |
|  | PDGFD -> AP-1 Expression Targets |
|  | PDGF -> AP-1/CREB/CREBBP/MYC Expression Targets |
|  | KIT -> MITF Signaling |
|  | PDGFR -> AP-1/MYC Signaling |
|  | AVP/Gs -> CREB/ELK/SRF/AP-1/EGR Expression Targets |
|  | GNRH1 Expression Targets |
|  | NTS Expression Targets |
|  | GAST Expression Targets in GAST Expression Targets |
|  | AGT -> TP53 Expression Targets |
|  | GNRH2 Expression Targets |
|  | OXT Expression Targets |
|  | POMC Expression Targets |
|  | CCL11 Expression Targets |
|  | EDN1 Expression Targets |
|  | AGT -> CREB Expression Targets |
|  | AVP/Gs -> MEF/MYOD/NFATC/MYOG Expression Targets |
|  | NPY Expression Targets |
|  | AVP/Gq -> STAT Expression Targets |
|  | IFNA1/Gq Expression Targets |
|  | AGT -> ELK/SRF Expression Targets |
|  | TAC1 Expression Targets |
|  | AVP/Gq -> MEF/MYOD/NFATC/MYOG Expression Targets |
|  | EDN3 Expression Targets |
|  | AVP/Gq -> CREB/ELK/SRF/AP-1/EGR Expression Targets |
|  | PDCD1 -> ATF/CREB/CREBBP Expression Targets |
|  | PDCD1 -> NFATC Expression Targets |
|  | PDCD1 -> AP-1 Expression Targets |
|  | PTPRC -> BCL6 Signaling |
|  | AngiopoietinR -> AP-1 Signaling |
|  | GDNF -> HSF1 Signaling |
|  | NTRK -> AP-1/CREB/ELK/SRF/MYC/SMAD3/TP53 Signaling |
|  | GAS6 -> AP-1/CREB Expression Targets |
|  | FIGF -> AP-1 Expression Target |
|  | FIGF -> NCOR2 Expression Target |
|  | KITLG -> AP-1/CREB/CREBBP/MYC Expression Targets |
|  | GDNF -> HSF1 Expression Targets |
|  | CSF1 -> AP-1/CREB/CREBBP/MYC Expression Targets |
|  | PGF -> AP-1/CREB/CREBBP/MYC Expression Targets |
|  | FLT3LG -> AP-1/CREB/CREBBP Expression Targets |
|  | WNT/Calcium non-Canonical Signaling |
|  | Lysophosphatidic Acid/LPARs Signaling |
|  | Ras-GRF Regulation Signaling |
|  | MERTK Signaling |
|  | Ras Signaling |
|  | AXL Receptor Tyrosine Kinase Signaling |
|  | VEGF Signaling |
|  | Estrogens/ESR1 non-Genomic Signaling |
|  | EGFR Signaling |
|  | NF-kB Canonical Signaling |
|  | ERK/MAPK Canonical Signaling |
|  | TCR -> CREB/CREBBP/ATF Expression Targets |
|  | TCR -> NF-kB Expression Targets |
|  | TCR -> NFAT Expression Targets |
|  | TCR -> AP-1 Expression Targets |
|  | T-Cell Receptor -> NF-kB Signaling |
|  | T-Cell Receptor -> NFATC Signaling |
|  | T-Cell Receptor -> CREBBP Signaling |
|  | TGFA -> TP53 Expression Targets |
|  | TGFA -> HIF1A Expression Targets |
|  | TGFA -> MEF/MYOD/NFATC Expression Targets |
|  | TGFA/AP-1/ATF Expression Targets |
|  | TGFA -> CREB/CREBBP/ELK-SRF/MYC Expression Targets |
|  | Thrombopoietin -> AP-1/CREB/CREBBP/MYC Expression Targets |
|  | IL1B -> PGE2 Expression Targets |
|  | Elevated Receptors -> Expression Targets in Kidney |
|  | PLAU -> ELK-SRF/AP-1 Expression Targets |
|  | VEGFA -> ATF/CREB/ELK-SRF Expression Targets |
|  | VEGFA -> NFATC Expression Targets |
|  | VEGFC -> ATF Expression Target |
|  | VEGFA -> AP-1/CREBBP/MYC Expression Targets |
|  | VEGFA -> NCOR2 Expression Target |
|  | VEGFR -> NFATC Signaling |
|  | VEGFR -> AP-1/CREB/MYC Signaling |
|  | VEGFR -> ATF/CREB/ELK-SRF Signaling |
|  | ace2 |
|  | cypa |
|  | Model of PPARG signaling in psoriasis |
|  | New Pathway |
|  | 2\_Acute phase initiation in atopic dermatitis |
|  | 1\_The onset of atopic dermatitis |
|  | 2\_Podocyte dysfunction in glomerulonephritis |
|  | 3\_Enhanced angiogenesis in endometriosis |
|  | 1\_Disruption of luteinizing hormone and follicle-stimulating hormone secretion in PCOS |
|  | 2\_Oxidative stress, all-trans-retinal, and lipofuscin toxicity in agerelated macular degeneration |
|  | 2\_Pathogens stimulate mucins expression in the middle ear |
|  | 2\_Oxidized LDL causes endothelial dysfunction in atherosclerosis |
|  | 3\_Dysfunction of smooth muscle cells in hypertension |
|  | 4\_Endothelial cells repress vasoconstriction in hypertension |
|  | 1\_Rise in aldosterone synthesis in adrenal zona glomerulosa cells |
|  | 2\_The intense proliferation and contractile activity of smooth muscle cells in pulmonary hypertension |
|  | 1\_Cigarette smoke causes dysfunction and production of inflammatory mediators in alveolar epithelial cells |
|  | 3\_1\_Mucus accumulation in asthma |
|  | 2\_1\_Eosinophilia and neutrophilia in asthma: eosinophil migration and activation |
|  | 2\_2\_Eosinophilia and neutrophilia in asthma: eosinophil apoptosis |
|  | 4\_1\_Airway smooth muscle cells contraction |
|  | 4\_2\_Airway smooth muscle cells proliferation and airway tissue remodeling |
|  | 4\_Mucin hyperproduction in goblet and mucous cells in COPD |
|  | 1\_CD4+ T cell and epithelial cell response in celiac disease |
|  | 2\_CD8+ T-cell response and mucosal damage in celiac disease |
|  | 1\_2\_Insulin synthesis and secretion: insulin secretion |
|  | 1\_1\_Insulin synthesis and secretion: insulin processing and storage |
|  | 1\_CFTR expression and degradation in epithelial cells in cystic fibrosis (class I-II mutations) |
|  | 3\_Islet beta-cell destruction in T1D |
|  | 3\_Pain perception in diabetic neuropathy |
|  | 2\_Neuron and Schwann cell death |
|  | 1\_1\_Glutamate overload enforced prostaglandins and NO synthesis in neurons |
|  | 1\_2\_Glutamate overload and NO-mediated vasodilation |
|  | 1\_Decreased secretion of thyroid hormones |
|  | 2\_1\_General pathways of thyroid hormone signaling |

---

|  |  |
| --- | --- |
| Source | curated |
|  | GO component |

---

|  |  |
| --- | --- |
| MedScan ID | 3708 |
|  | 622741 |

---

|  |  |
| --- | --- |
| LocusLink ID | 3708 |
|  | 16438 |
|  | 25262 |
|  | 100038404 |
|  | 18544 |
|  | 317697 |
|  | 567611 |
|  | 57043 |
|  | 140449 |

---

|  |  |
| --- | --- |
| Alias | Purkinje cell protein 1 |
|  | OTTHUMP00000209578 |
|  | Ins(1,4,5)P3 receptor I |
|  | IP3R I |
|  | D6Pas2 |
|  | ITPR1 |
|  | OTTHUMP00000207085 |
|  | SCA29 locus |
|  | SCA16 protein |
|  | spinocerebellar ataxia 16 protein |
|  | Pcp1 |
|  | SCA29 gene |
|  | opisthotonus gene |
|  | SCA16 locus |
|  | protein PCD-6 |
|  | Gm10429 |
|  | InsP3R1 |
|  | DKFZp313E1334 |
|  | Purkinje cell protein I |
|  | SCA16 gene |
|  | SCA16 region |
|  | Inositol 1,4,5-trisphosphate receptor 1 |
|  | inositol 1,4,5-triphosphate receptor I |
|  | Inositol 1,4,5-trisphosphate receptor I |
|  | SCA29 region |
|  | InsP3R |
|  | type 1 InsP3 receptor |
|  | SCA29 protein |
|  | InsP3R I |
|  | SCA15 gene |
|  | PPP1R94 |
|  | inositol 1,4,5-trisphosphate-binding protein P400 |
|  | ENSMUSG00000072853 |
|  | SCA15 locus |
|  | spinocerebellar ataxia-16 protein |
|  | Pcp I |
|  | ITPR I |
|  | IP3R1 |
|  | protein phosphatase 1, regulatory subunit 94 |
|  | SCA15 region |
|  | spinocerebellar ataxia-29 gene |
|  | inositol 145-triphosphate receptor I |
|  | InsP>3<R type I |
|  | spinocerebellar ataxia 15 protein |
|  | DKFZp313N1434 |
|  | type 1 inositol 1,4,5-trisphosphate receptor |
|  | inositol 145-triphosphate receptor 1 |
|  | spinocerebellar ataxia 29 protein |
|  | SCA15 protein |
|  | inositol 1,4,5-triphosphate receptor 1 |
|  | spinocerebellar ataxia-29 protein |
|  | spinocerebellar ataxia 29 gene |
|  | spinocerebellar ataxia-15 protein |
|  | Ins(1,4,5)P3 receptor 1 |
|  | I145TR |
|  | ACV |
|  | CLA4 |
|  | IP3R |
|  | SCA15 |
|  | SCA16 |
|  | SCA29 |
|  | IP3 receptor |
|  | IP3R 1 |
|  | inositol 1,4,5-triphosphate receptor, type 1 |
|  | inositol 1,4,5-trisphosphate receptor type 1 |
|  | opt |
|  | P400 |
|  | wblo |
|  | Pcp-1 |
|  | Itpr-1 |
|  | opisthotonus |
|  | IP-3-R |
|  | spinocerebellar ataxia 15 |
|  | Spinocerebellar ataxia 29 |
|  | spinocerebellar ataxia 16 |
|  | inositol 1,4,5-trisphosphate receptor, type 1 |
|  | InsP3R type I |
|  | inositol 145-triphosphate receptor type 1 |
|  | inositol 1,4,5-triphosphate receptor type 1 |
|  | inositol 145-triphosphate receptor type I |
|  | inositol 1,4,5-triphosphate receptor, type |
|  | InsP>3<R type 1 |
|  | Inositol 1,4,5-trisphosphate receptor type I |

---

|  |  |
| --- | --- |
| GO ID | 0019855 |
|  | 0005509 |
|  | 0015085 |
|  | 0015278 |
|  | 0070679 |
|  | 0098695 |
|  | 0005220 |
|  | 0035091 |
|  | 0006816 |
|  | 0032469 |
|  | 0042045 |
|  | 0048016 |
|  | 0070059 |
|  | 0050849 |
|  | 0030168 |
|  | 0009791 |
|  | 0010506 |
|  | 1903779 |
|  | 0050796 |
|  | 0099566 |
|  | 0051209 |
|  | 0001666 |
|  | 0007165 |
|  | 0050882 |
|  | 0098685 |
|  | 0005955 |
|  | 0030659 |
|  | 0005783 |
|  | 0005789 |
|  | 0016021 |
|  | 0016020 |
|  | 0005637 |
|  | 0005730 |
|  | 0048471 |
|  | 0005886 |
|  | 0031088 |
|  | 0031094 |
|  | 0031095 |
|  | 0014069 |
|  | 0016529 |
|  | 0030667 |
|  | 0030658 |
|  | 0005262 |
|  | 0005216 |
|  | 0008022 |
|  | 0019903 |
|  | 0044877 |
|  | 0006915 |
|  | 0070588 |
|  | 0071320 |
|  | 0006811 |
|  | 0097421 |
|  | 1901215 |
|  | 0043065 |
|  | 0051928 |
|  | 0007204 |
|  | 2000347 |
|  | 0010976 |
|  | 0051289 |
|  | 0055085 |
|  | 0098982 |
|  | 0005737 |
|  | 0031410 |
|  | 0030425 |
|  | 0030176 |
|  | 0043231 |
|  | 0045121 |
|  | 0043025 |
|  | 0005635 |
|  | 0098794 |
|  | 0098793 |
|  | 0032991 |
|  | 0030868 |
|  | 0097060 |
|  | 0071456 |
|  | 0016358 |
|  | 0032403 |
|  | 0006810 |
|  | 0005829 |
|  | 0043234 |
|  | 0005218 |
|  | 0038095 |
|  | 0038096 |
|  | 0007202 |
|  | 0007596 |
|  | 0006112 |
|  | 0007173 |
|  | 0008543 |
|  | 0045087 |
|  | 0048011 |
|  | 0044281 |
|  | 0048010 |
|  | 0005623 |
|  | 0005622 |
|  | 0008219 |
|  | 0008095 |
|  | 0004872 |
|  | 0034220 |
|  | 0005792 |
|  | 0005515 |
|  | 0005887 |
|  | 0006812 |
|  | 0005261 |

---

|  |  |
| --- | --- |
| KEGG ID | hsa:3708 |
|  | mmu:16438 |
|  | rno:25262 |

---

|  |  |
| --- | --- |
| MedLine Reference | 11316737 |
|  | 11587548 |
|  | 11972451 |
|  | 12143036 |
|  | 12167631 |
|  | 12435588 |
|  | 12477932 |
|  | 12623131 |
|  | 12820984 |
|  | 1338970 |
|  | 14517800 |
|  | 14982933 |
|  | 15060019 |
|  | 15364918 |
|  | 15739177 |
|  | 15774532 |
|  | 15843050 |
|  | 15911880 |
|  | 16118475 |
|  | 16223514 |
|  | 16237118 |
|  | 16377004 |
|  | 16691292 |
|  | 16723353 |
|  | 16840702 |
|  | 1849282 |
|  | 2165071 |
|  | 8567977 |
|  | 9073177 |
|  | 10051594 |
|  | 10095099 |
|  | 10098882 |
|  | 10349636 |
|  | 10426189 |
|  | 10482245 |
|  | 10505685 |
|  | 10609087 |
|  | 10811729 |
|  | 10922068 |
|  | 11040263 |
|  | 11042159 |
|  | 11076861 |
|  | 11172053 |
|  | 11217851 |
|  | 11334652 |
|  | 11368972 |
|  | 11476286 |
|  | 11696558 |
|  | 11700294 |
|  | 11860456 |
|  | 11955285 |
|  | 11980893 |
|  | 12110570 |
|  | 12118121 |
|  | 12421829 |
|  | 12442173 |
|  | 12444087 |
|  | 12466851 |
|  | 12611586 |
|  | 12621039 |
|  | 12654335 |
|  | 12828938 |
|  | 12967979 |
|  | 12972155 |
|  | 1374893 |
|  | 14506248 |
|  | 14687547 |
|  | 14706453 |
|  | 14722080 |
|  | 14741211 |
|  | 15016804 |
|  | 15026142 |
|  | 15184066 |
|  | 15201137 |
|  | 15284241 |
|  | 15583010 |
|  | 15590059 |
|  | 15652484 |
|  | 15664189 |
|  | 15890645 |
|  | 16014380 |
|  | 16091359 |
|  | 16141072 |
|  | 16141073 |
|  | 16286649 |
|  | 16458354 |
|  | 1648733 |
|  | 16606357 |
|  | 1662017 |
|  | 16675011 |
|  | 1672287 |
|  | 16731645 |
|  | 16783635 |
|  | 16793548 |
|  | 16844763 |
|  | 16925983 |
|  | 16964447 |
|  | 17038520 |
|  | 17050730 |
|  | 2554142 |
|  | 2707487 |
|  | 2762133 |
|  | 3141586 |
|  | 3199205 |
|  | 7642703 |
|  | 7755592 |
|  | 7852357 |
|  | 7896292 |
|  | 7945203 |
|  | 7946968 |
|  | 8081710 |
|  | 8530061 |
|  | 8538767 |
|  | 8641130 |
|  | 8661054 |
|  | 8758947 |
|  | 8780003 |
|  | 8863127 |
|  | 8900188 |
|  | 8930970 |
|  | 8987786 |
|  | 9124414 |
|  | 9231705 |
|  | 9302515 |
|  | 9615221 |
|  | 9697703 |
|  | 9808793 |
|  | 9858485 |
|  | 9867846 |
|  | 9867847 |
|  | 9986730 |
|  | 10209024 |
|  | 10724174 |
|  | 10843712 |
|  | 10874040 |
|  | 11163362 |
|  | 11524429 |
|  | 11574661 |
|  | 11782428 |
|  | 12032348 |
|  | 12196544 |
|  | 12356940 |
|  | 12480535 |
|  | 12525476 |
|  | 12529267 |
|  | 12606542 |
|  | 12676536 |
|  | 12717445 |
|  | 12754204 |
|  | 12766172 |
|  | 14570872 |
|  | 14635192 |
|  | 14685260 |
|  | 14697242 |
|  | 14707143 |
|  | 14761954 |
|  | 15067324 |
|  | 15104175 |
|  | 15613488 |
|  | 15623527 |
|  | 16223735 |
|  | 16621795 |
|  | 17130290 |
|  | 7500840 |
|  | 7559486 |
|  | 8132516 |
|  | 8132598 |
|  | 8521476 |
|  | 8648241 |
|  | 8713116 |
|  | 9346894 |
|  | 9511764 |
|  | 9607712 |
|  | 9808459 |
|  | 9853757 |
|  | 11584008 |
|  | 12714606 |
|  | 2174422 |
|  | 10737800 |
|  | 15146197 |
|  | 16344560 |
|  | 17081983 |
|  | 17256008 |
|  | 17437169 |
|  | 17590087 |
|  | 11544199 |
|  | 15489334 |
|  | 16527252 |
|  | 17208939 |
|  | 17327232 |
|  | 17416589 |
|  | 17482902 |
|  | 17511035 |
|  | 9651219 |
|  | 10506212 |
|  | 12873381 |
|  | 16103111 |
|  | 17284437 |
|  | 17496801 |
|  | 17636122 |
|  | 11445634 |
|  | 17030759 |
|  | 11723290 |
|  | 14981189 |

---

|  |  |
| --- | --- |
| Organism | Homo sapiens {Organism urn:agi-taxid:9606} |
|  | Mus musculus {Organism urn:agi-taxid:10090} |
|  | Rattus norvegicus {Organism urn:agi-taxid:10116} |
|  | Homo sapiens |
|  | Mus musculus |
|  | Rattus norvegicus |
|  | Bos taurus |
|  | Danio rerio |

---

|  |  |
| --- | --- |
| Mouse chromosome position | 6 49.74 cM |
|  | 6 48.0 cM |

---

|  |  |
| --- | --- |
| OMIM ID | 147265 |
|  | 206700 |
|  | 606658 |
|  | 117360 |
|  | 606364 |

---

|  |  |
| --- | --- |
| Rat chromosome position | 4q41 |

---

|  |  |
| --- | --- |
| Hugo ID | 6180 |
|  | HGNC:6180 |
|  | 10554 |
|  | 13439 |

---

|  |  |
| --- | --- |
| Human chromosome position | 3p26.1 |
|  | 3p26-p25 |

---

|  |  |
| --- | --- |
| Swiss-Prot Accession | B4DER3 |
|  | Q14643 |
|  | Q59H91 |
|  | B4DGH1 |
|  | Q14643.3 |
|  | P11881 |
|  | Q3UVA1 |
|  | Q8BQN0 |
|  | Q8C7X9 |
|  | Q8C8N0 |
|  | P11881.2 |
|  | C7E1V2 |
|  | Q5BJS8 |
|  | F1LQX8 |
|  | A0A0A0MY31 |
|  | P29994 |
|  | P29994.2 |
|  | E7EPX7 |
|  | E9PDE9 |
|  | Q14660 |
|  | Q99897 |
|  | P20943 |
|  | Q99LG5 |
|  | Q62869 |
|  | Q6NV80 |
|  | ITPR1\_HUMAN |
|  | ITPR1\_RAT |
|  | Q8BQN0\_MOUSE |

---

|  |  |
| --- | --- |
| PIR ID | A55713 |
|  | S54974 |
|  | S04844 |
|  | A36579 |
|  | B36579 |

---

|  |  |
| --- | --- |
| GenBank ID | NC\_000003 |
|  | NM\_001099952 |
|  | NP\_001093422 |
|  | NM\_001168272 |
|  | NP\_001161744 |
|  | NM\_002222 |
|  | NP\_002213 |
|  | XM\_011533681 |
|  | XP\_011531983 |
|  | XM\_011533683 |
|  | XP\_011531985 |
|  | XM\_011533685 |
|  | XP\_011531987 |
|  | XM\_011533686 |
|  | XP\_011531988 |
|  | XM\_006713131 |
|  | XP\_006713194 |
|  | XM\_005265109 |
|  | XP\_005265166 |
|  | XM\_005265110 |
|  | XP\_005265167 |
|  | XM\_011533684 |
|  | XP\_011531986 |
|  | XM\_011533687 |
|  | XP\_011531989 |
|  | XM\_011533688 |
|  | XP\_011531990 |
|  | XM\_011533690 |
|  | XP\_011531992 |
|  | XM\_017006357 |
|  | XP\_016861846 |
|  | XM\_011533691 |
|  | XP\_011531993 |
|  | XM\_011533692 |
|  | XP\_011531994 |
|  | XM\_017006358 |
|  | XP\_016861847 |
|  | XM\_011533682 |
|  | XP\_011531984 |
|  | NG\_016144 |
|  | AC018816 |
|  | AC024168 |
|  | AC069248 |
|  | AC090944 |
|  | AF009963 |
|  | AL832689 |
|  | AL832701 |
|  | CH471055 |
|  | EAW63908 |
|  | EAW63909 |
|  | EAW63910 |
|  | EAW63911 |
|  | EAW63912 |
|  | EAW63913 |
|  | EAW63914 |
|  | EAW63915 |
|  | KF457534 |
|  | KF457537 |
|  | KF457539 |
|  | KF457541 |
|  | KF457546 |
|  | KF495812 |
|  | L47220 |
|  | AB208868 |
|  | BAD92105 |
|  | AI470158 |
|  | AK293752 |
|  | BAG57174 |
|  | AK294594 |
|  | BAG57782 |
|  | AK296235 |
|  | BAG58955 |
|  | AK309981 |
|  | AK311008 |
|  | AW501800 |
|  | BC144563 |
|  | AAI44564 |
|  | BG202086 |
|  | BM695661 |
|  | BQ006717 |
|  | CN431631 |
|  | CV355426 |
|  | D26070 |
|  | BAA05065 |
|  | DA060305 |
|  | DB260851 |
|  | DB544988 |
|  | DB576768 |
|  | L38019 |
|  | AAB04947 |
|  | S82269 |
|  | AAD14386 |
|  | U23850 |
|  | Q14643 |
|  | NC\_000072 |
|  | XM\_006505631 |
|  | XP\_006505694 |
|  | XM\_006505623 |
|  | XP\_006505686 |
|  | XM\_017321407 |
|  | XP\_017176896 |
|  | XM\_006505627 |
|  | XP\_006505690 |
|  | XM\_006505624 |
|  | XP\_006505687 |
|  | XM\_006505625 |
|  | XP\_006505688 |
|  | XM\_006505632 |
|  | XP\_006505695 |
|  | XM\_006505628 |
|  | XP\_006505691 |
|  | XM\_006505629 |
|  | XP\_006505692 |
|  | XM\_006505633 |
|  | XP\_006505696 |
|  | XM\_017321408 |
|  | XP\_017176897 |
|  | XM\_006505636 |
|  | XP\_006505699 |
|  | XM\_006505626 |
|  | XP\_006505689 |
|  | XM\_017321409 |
|  | XP\_017176898 |
|  | XM\_006505630 |
|  | XP\_006505693 |
|  | XM\_017321410 |
|  | XP\_017176899 |
|  | XM\_006505637 |
|  | XP\_006505700 |
|  | NM\_010585 |
|  | NP\_034715 |
|  | AC120411 |
|  | AC153986 |
|  | AC156506 |
|  | AH001976 |
|  | AAA39316 |
|  | AAA39317 |
|  | AY857958 |
|  | CH466523 |
|  | EDK99408 |
|  | EDK99409 |
|  | D78171 |
|  | AK042240 |
|  | AK044776 |
|  | BAC32085 |
|  | AK047719 |
|  | BAC33138 |
|  | AK049015 |
|  | BAC33514 |
|  | AK083514 |
|  | AK087694 |
|  | AK137477 |
|  | BAE23370 |
|  | BC003271 |
|  | AAH03271 |
|  | BC068269 |
|  | AAH68269 |
|  | BQ180028 |
|  | BU705754 |
|  | BX633137 |
|  | BY145967 |
|  | CB521690 |
|  | CF168791 |
|  | CF539692 |
|  | CF744794 |
|  | CJ236273 |
|  | CK636937 |
|  | CN459019 |
|  | CN692248 |
|  | DN176734 |
|  | DV652411 |
|  | M21530 |
|  | AAA88319 |
|  | S75867 |
|  | X15373 |
|  | CAA33433 |
|  | P11881 |
|  | NC\_005103 |
|  | XM\_006236987 |
|  | XP\_006237049 |
|  | NM\_001007235 |
|  | NP\_001007236 |
|  | NM\_001270597 |
|  | NP\_001257526 |
|  | NM\_001270596 |
|  | NP\_001257525 |
|  | XM\_008763167 |
|  | XP\_008761389 |
|  | XM\_008763168 |
|  | XP\_008761390 |
|  | XM\_008763171 |
|  | XP\_008761393 |
|  | XM\_008763172 |
|  | XP\_008761394 |
|  | XM\_008763174 |
|  | XP\_008761396 |
|  | XM\_008763176 |
|  | XP\_008761398 |
|  | XM\_008763169 |
|  | XP\_008761391 |
|  | XM\_008763170 |
|  | XP\_008761392 |
|  | XM\_008763173 |
|  | XP\_008761395 |
|  | XM\_008763177 |
|  | XP\_008761399 |
|  | XM\_008763175 |
|  | XP\_008761397 |
|  | XM\_008763178 |
|  | XP\_008761400 |
|  | AC\_000072 |
|  | AABR07061705 |
|  | AABR07061706 |
|  | AABR07061707 |
|  | AAHX01032183 |
|  | AAHX01032184 |
|  | AAHX01032185 |
|  | AAHX01032186 |
|  | AAHX01032187 |
|  | AAHX01032188 |
|  | CH473957 |
|  | EDL91478 |
|  | BC091346 |
|  | AAH91346 |
|  | BF402715 |
|  | FQ225812 |
|  | FQ233552 |
|  | GQ233032 |
|  | ACT21453 |
|  | J05510 |
|  | AAA41357 |
|  | AAA41358 |
|  | M64698 |
|  | AAA41448 |
|  | M64699 |
|  | AAA41447 |
|  | U38653 |
|  | AAC53099 |
|  | U38665 |
|  | AAB51330 |
|  | U38812 |
|  | AAC53100 |
|  | P29994 |
|  | XM\_011533689 |
|  | XP\_011531991 |
|  | NC\_018914 |
|  | AMYH02006182 |
|  | AMYH02006183 |
|  | AMYH02006184 |
|  | XM\_006505635 |
|  | XP\_006505698 |
|  | XM\_006505634 |
|  | XP\_006505697 |
|  | AC\_000028 |
|  | AAHY01056234 |
|  | AAHY01056235 |
|  | AAHY01056236 |
|  | AAHY01056237 |
|  | AAHY01056238 |
|  | AAHY01056239 |
|  | AAHY01056240 |
|  | AAHY01056241 |
|  | AAHY01056242 |
|  | AAHY01056243 |
|  | AAHY01056244 |
|  | AAHY01056245 |
|  | AAHY01056246 |
|  | AAHY01056247 |
|  | AAHY01056248 |
|  | AAHY01056249 |
|  | BC172648 |
|  | AAI72648 |
|  | XM\_006713136 |
|  | XP\_006713199 |
|  | XM\_006713135 |
|  | XP\_006713198 |
|  | XM\_006713132 |
|  | XP\_006713195 |
|  | XM\_006713133 |
|  | XP\_006713196 |
|  | XM\_006713134 |
|  | XP\_006713197 |
|  | AC\_000135 |
|  | ABBA01020466 |
|  | ABBA01020467 |
|  | ABBA01020468 |
|  | ABBA01020469 |
|  | ABBA01020470 |
|  | M75986 |
|  | M75987 |
|  | XM\_005265108 |
|  | XP\_005265165 |
|  | XM\_006236989 |
|  | XP\_006237051 |
|  | XM\_006236990 |
|  | XP\_006237052 |
|  | XM\_006236993 |
|  | XP\_006237055 |
|  | XM\_006236988 |
|  | XP\_006237050 |
|  | XM\_006236991 |
|  | XP\_006237053 |
|  | XM\_006236992 |
|  | XP\_006237054 |
|  | XM\_006236994 |
|  | XP\_006237056 |
|  | AABR06032821 |
|  | AABR06032822 |
|  | AABR06032823 |
|  | AABR06032824 |
|  | AABR06032825 |
|  | NT\_022517 |
|  | NW\_921651 |
|  | NW\_001838876 |
|  | AC\_000046 |
|  | Q59H91 |
|  | NT\_039353 |
|  | NW\_001030811 |
|  | Q3UVA1 |
|  | Q6NV80 |
|  | Q8BQN0 |
|  | Q8C7X9 |
|  | Q8C8N0 |
|  | NW\_047695 |
|  | NW\_001084830 |
|  | Q5BJS8 |
|  | Q8C9F0 |
|  | NW\_000262 |
|  | BG068206 |
|  | 1054963 |
|  | 1055287 |
|  | 1098969 |
|  | 17380349 |
|  | 204674 |
|  | 204675 |
|  | 204967 |
|  | 204969 |
|  | 60688455 |
|  | 81882514 |
|  | 1196648 |
|  | 123788591 |
|  | 129358 |
|  | 13096949 |
|  | 198412 |
|  | 26336805 |
|  | 26338934 |
|  | 26339686 |
|  | 46189240 |
|  | 53569 |
|  | 554171 |
|  | 74210346 |
|  | 81874734 |
|  | 81877206 |
|  | 81892040 |
|  | 81899258 |
|  | 81899351 |
|  | 17366467 |
|  | 4262086 |
|  | 46107962 |
|  | 559323 |
|  | 62087316 |
|  | 74741363 |
|  | NT\_086636 |
|  | 17367101 |
|  | 6006803 |
|  | AAF00613 |
|  | AF157625 |
|  | NC\_007320 |
|  | NM\_174841 |
|  | NP\_777266 |
|  | NW\_001494089 |
|  | Q9TU34 |
|  | NC\_007117 |
|  | NW\_001513595 |
|  | XM\_690915 |
|  | XP\_696007 |
|  | 119584312 |
|  | 119584313 |
|  | 119584314 |
|  | 119584315 |
|  | 119584316 |
|  | 119584317 |
|  | 119584318 |
|  | 119584319 |
|  | 148666992 |
|  | 148666993 |
|  | 149036860 |

---

|  |  |
| --- | --- |
| Swiss-Prot ID | ITPR1\_HUMAN |
|  | ITPR1\_MOUSE |
|  | ITPR1\_RAT |
|  | B4DER3\_HUMAN |
|  | Q59H91\_HUMAN |
|  | E9PDE9\_HUMAN |
|  | B4DGH1\_HUMAN |
|  | Q8BQN0\_MOUSE |
|  | Q8C8N0\_MOUSE |
|  | Q8C7X9\_MOUSE |
|  | Q3UVA1\_MOUSE |

---

|  |  |
| --- | --- |
| Cell Localization | Endoplasmic reticulum membrane |
|  | Cytoplasmic vesicle |
|  | Secretory vesicle membrane |
|  | Cytoplasm |
|  | Perinuclear region |
|  | Endoplasmic reticulum |
|  | Plasma membrane |

---

|  |  |
| --- | --- |
| Ensembl ID | ENSG00000150995 |
|  | ENSP00000349597.4 |
|  | ENST00000357086.10 |
|  | ENSP00000306253.8 |
|  | ENST00000302640.12 |
|  | ENSP00000397885.2 |
|  | ENST00000456211.8 |
|  | ENSP00000497605.1 |
|  | ENST00000649015.1 |
|  | ENSP00000346595.8 |
|  | ENST00000354582.11 |
|  | ENSP00000498056.1 |
|  | ENST00000650294.1 |
|  | ENSP00000497026.1 |
|  | ENST00000648309.1 |
|  | ENSMUSG00000030102 |
|  | ENSMUSP00000032192.6 |
|  | ENSMUST00000032192.8 |
|  | ENSMUSP00000144880.1 |
|  | ENSMUST00000203615.2 |
|  | ENSRNOG00000007104 |
|  | ENSRNOP00000063885.3 |
|  | ENSRNOT00000064025.3 |
|  | ENSRNOP00000049540.4 |
|  | ENSRNOT00000041130.5 |
|  | ENSRNOP00000048661.4 |
|  | ENSRNOT00000043646.4 |
|  | ENSRNOP00000075403.1 |
|  | ENSRNOT00000082723.1 |
|  | ENSP00000349597 |
|  | ENST00000357086 |
|  | ENSP00000306253 |
|  | ENST00000302640 |
|  | ENSP00000397885 |
|  | ENST00000456211 |
|  | ENSP00000346595 |
|  | ENST00000354582 |
|  | ENSMUSP00000032192 |
|  | ENSMUST00000032192 |
|  | ENSRNOP00000063885 |
|  | ENSRNOT00000064025 |
|  | ENSRNOP00000049540 |
|  | ENSRNOT00000041130 |
|  | ENSRNOP00000048661 |
|  | ENSRNOT00000043646 |
|  | ENSRNOP00000043615 |
|  | ENSRNOT00000040255 |

---

|  |  |
| --- | --- |
| MGI ID | MGI:96623 |
|  | 96623 |
|  | 97507 |

---

|  |  |
| --- | --- |
| RGD ID | 2933 |

---

|  |  |
| --- | --- |
| TCDB ID | 1.A.3.2.6 |

---

|  |  |
| --- | --- |
| Unigene ID | Hs.567295 |
|  | Hs.715765 |
|  | Mm.227912 |
|  | Rn.2135 |
|  | Rn.170703 |
|  | Hs.708300 |
|  | Bt.22305 |
|  | Dr.115352 |
|  | Hs.699169 |

---

|  |  |
| --- | --- |
| Homologene ID | 1673 |

---

|  |  |
| --- | --- |
| Shape | III-vertex |

---

|  |  |
| --- | --- |
| IPI ID | IPI00230873 |
|  | IPI00230877 |
|  | IPI00230879 |
|  | IPI00230881 |
|  | IPI00944825 |
|  | IPI00947857 |
|  | IPI00949472 |
|  | IPI00950591 |
|  | IPI00218658 |
|  | IPI00218659 |
|  | IPI00218660 |
|  | IPI00218661 |
|  | IPI00218662 |
|  | IPI00218664 |
|  | IPI00333753 |
|  | IPI00925443 |
|  | IPI00117630 |
|  | IPI00230018 |
|  | IPI00230019 |
|  | IPI00230020 |
|  | IPI00230021 |
|  | IPI00230023 |
|  | IPI00469221 |
|  | IPI00474081 |
|  | IPI00982630 |
|  | IPI00218663 |
|  | IPI00795991 |
|  | IPI00230875 |
|  | IPI00210027 |
|  | IPI00230878 |
|  | IPI00230880 |

---

|  |  |
| --- | --- |
| Microarray ID | 161661\_i\_at |
|  | 93895\_s\_at |
|  | 94977\_at |
|  | 240052\_at |

---

|  |  |
| --- | --- |
| Homo sapiens Chromosome position | 3p26-p25 |
|  | 8q23-q24.1 |
|  | 3pter-p24.2 |

---

|  |  |
| --- | --- |
| Ensembl Transcript ID | ENST00000357086 |

---

|  |  |
| --- | --- |
| ENSEMBL | ENSG00000150995 |

---

|  |  |
| --- | --- |
| FunctionalClass | calcium ion transporter |
|  | inositol-1,4,5-triphosphate receptor |
|  | protein binding |
|  | calcium channel |
|  | calcium ion binding |
|  | inositol 1,4,5-triphosphate-sensitive calcium-release channel |
|  | ion channel |
|  | receptor |
|  | cation channel |
|  | calcium ion transmembrane transporter |

---

|  |  |
| --- | --- |
| Bos taurus Chromosome position | 22 |

---

|  |  |
| --- | --- |
| Mus musculus Chromosome position | 6 48.0 cM |
|  | 6 45.0 cM |

---

|  |  |
| --- | --- |
| KEGG pathway | Calcium signaling pathway |
|  | Gap junction |
|  | GnRH signaling pathway |
|  | Long-term depression |
|  | Long-term potentiation |
|  | Phosphatidylinositol signaling system |

---

|  |  |
| --- | --- |
| ProteinAtlas SubcellularLocation | Main location: Cytoplasm;Cytoskeleton (Intermediate filaments); Other location: ; Expression type: APE; Reliability: Very low |

---

|  |  |
| --- | --- |
| Rattus norvegicus Chromosome position | 4q41 |

---

|  |  |
| --- | --- |
| Ensembl Gene ID | ENSG00000150995 |

---

|  |  |
| --- | --- |
| ProteinAtlas NormalTissue | Tissue: adrenal gland; Cell type: glandular cells; Level: None; Expression type: APE; Reliability: Medium |
|  | Tissue: appendix; Cell type: glandular cells; Level: None; Expression type: APE; Reliability: Medium |
|  | Tissue: appendix; Cell type: lymphoid tissue; Level: None; Expression type: APE; Reliability: Medium |
|  | Tissue: bone marrow; Cell type: hematopoietic cells; Level: None; Expression type: APE; Reliability: Medium |
|  | Tissue: breast; Cell type: glandular cells; Level: Low; Expression type: APE; Reliability: Medium |
|  | Tissue: bronchus; Cell type: respiratory epithelial cells; Level: None; Expression type: APE; Reliability: Medium |
|  | Tissue: cerebellum; Cell type: cells in granular layer; Level: None; Expression type: APE; Reliability: Medium |
|  | Tissue: cerebellum; Cell type: cells in molecular layer; Level: None; Expression type: APE; Reliability: Medium |
|  | Tissue: cerebellum; Cell type: Purkinje cells; Level: High; Expression type: APE; Reliability: Medium |
|  | Tissue: cerebral cortex; Cell type: glial cells; Level: None; Expression type: APE; Reliability: Medium |
|  | Tissue: cerebral cortex; Cell type: neuronal cells; Level: Medium; Expression type: APE; Reliability: Medium |
|  | Tissue: cervix, uterine; Cell type: glandular cells; Level: None; Expression type: APE; Reliability: Medium |
|  | Tissue: cervix, uterine; Cell type: squamous epithelial cells; Level: None; Expression type: APE; Reliability: Medium |
|  | Tissue: colon; Cell type: glandular cells; Level: None; Expression type: APE; Reliability: Medium |
|  | Tissue: duodenum; Cell type: glandular cells; Level: None; Expression type: APE; Reliability: Medium |
|  | Tissue: epididymis; Cell type: glandular cells; Level: Low; Expression type: APE; Reliability: Medium |
|  | Tissue: esophagus; Cell type: squamous epithelial cells; Level: None; Expression type: APE; Reliability: Medium |
|  | Tissue: fallopian tube; Cell type: glandular cells; Level: None; Expression type: APE; Reliability: Medium |
|  | Tissue: gall bladder; Cell type: glandular cells; Level: None; Expression type: APE; Reliability: Medium |
|  | Tissue: heart muscle; Cell type: myocytes; Level: Low; Expression type: APE; Reliability: Medium |
|  | Tissue: hippocampus; Cell type: glial cells; Level: None; Expression type: APE; Reliability: Medium |
|  | Tissue: hippocampus; Cell type: neuronal cells; Level: Medium; Expression type: APE; Reliability: Medium |
|  | Tissue: kidney; Cell type: cells in glomeruli; Level: None; Expression type: APE; Reliability: Medium |
|  | Tissue: kidney; Cell type: cells in tubules; Level: Low; Expression type: APE; Reliability: Medium |
|  | Tissue: lateral ventricle; Cell type: glial cells; Level: None; Expression type: APE; Reliability: Medium |
|  | Tissue: lateral ventricle; Cell type: neuronal cells; Level: Medium; Expression type: APE; Reliability: Medium |
|  | Tissue: liver; Cell type: bile duct cells; Level: None; Expression type: APE; Reliability: Medium |
|  | Tissue: liver; Cell type: hepatocytes; Level: None; Expression type: APE; Reliability: Medium |
|  | Tissue: lung; Cell type: macrophages; Level: Low; Expression type: APE; Reliability: Medium |
|  | Tissue: lung; Cell type: pneumocytes; Level: None; Expression type: APE; Reliability: Medium |
|  | Tissue: lymph node; Cell type: germinal center cells; Level: Low; Expression type: APE; Reliability: Medium |
|  | Tissue: lymph node; Cell type: non-germinal center cells; Level: Low; Expression type: APE; Reliability: Medium |
|  | Tissue: nasopharynx; Cell type: respiratory epithelial cells; Level: None; Expression type: APE; Reliability: Medium |
|  | Tissue: oral mucosa; Cell type: squamous epithelial cells; Level: None; Expression type: APE; Reliability: Medium |
|  | Tissue: ovary; Cell type: ovarian stroma cells; Level: None; Expression type: APE; Reliability: Medium |
|  | Tissue: pancreas; Cell type: exocrine glandular cells; Level: None; Expression type: APE; Reliability: Medium |
|  | Tissue: pancreas; Cell type: islets of Langerhans; Level: None; Expression type: APE; Reliability: Medium |
|  | Tissue: parathyroid gland; Cell type: glandular cells; Level: None; Expression type: APE; Reliability: Medium |
|  | Tissue: placenta; Cell type: decidual cells; Level: None; Expression type: APE; Reliability: Medium |
|  | Tissue: placenta; Cell type: trophoblastic cells; Level: None; Expression type: APE; Reliability: Medium |
|  | Tissue: prostate; Cell type: glandular cells; Level: Low; Expression type: APE; Reliability: Medium |
|  | Tissue: rectum; Cell type: glandular cells; Level: None; Expression type: APE; Reliability: Medium |
|  | Tissue: salivary gland; Cell type: glandular cells; Level: None; Expression type: APE; Reliability: Medium |
|  | Tissue: seminal vesicle; Cell type: glandular cells; Level: Low; Expression type: APE; Reliability: Medium |
|  | Tissue: skeletal muscle; Cell type: myocytes; Level: None; Expression type: APE; Reliability: Medium |
|  | Tissue: skin; Cell type: epidermal cells; Level: None; Expression type: APE; Reliability: Medium |
|  | Tissue: small intestine; Cell type: glandular cells; Level: None; Expression type: APE; Reliability: Medium |
|  | Tissue: smooth muscle; Cell type: smooth muscle cells; Level: None; Expression type: APE; Reliability: Medium |
|  | Tissue: spleen; Cell type: cells in red pulp; Level: Low; Expression type: APE; Reliability: Medium |
|  | Tissue: spleen; Cell type: cells in white pulp; Level: None; Expression type: APE; Reliability: Medium |
|  | Tissue: stomach, lower; Cell type: glandular cells; Level: Low; Expression type: APE; Reliability: Medium |
|  | Tissue: stomach, upper; Cell type: glandular cells; Level: Low; Expression type: APE; Reliability: Medium |
|  | Tissue: testis; Cell type: cells in seminiferus ducts; Level: Low; Expression type: APE; Reliability: Medium |
|  | Tissue: testis; Cell type: Leydig cells; Level: Low; Expression type: APE; Reliability: Medium |
|  | Tissue: thyroid gland; Cell type: glandular cells; Level: None; Expression type: APE; Reliability: Medium |
|  | Tissue: tonsil; Cell type: germinal center cells; Level: None; Expression type: APE; Reliability: Medium |
|  | Tissue: tonsil; Cell type: non-germinal center cells; Level: Low; Expression type: APE; Reliability: Medium |
|  | Tissue: tonsil; Cell type: squamous epithelial cells; Level: None; Expression type: APE; Reliability: Medium |
|  | Tissue: urinary bladder; Cell type: urothelial cells; Level: None; Expression type: APE; Reliability: Medium |
|  | Tissue: uterus, post-menopause; Cell type: cells in endometrial stroma; Level: None; Expression type: APE; Reliability: Medium |
|  | Tissue: uterus, post-menopause; Cell type: glandular cells; Level: None; Expression type: APE; Reliability: Medium |
|  | Tissue: uterus, pre-menopause; Cell type: cells in endometrial stroma; Level: None; Expression type: APE; Reliability: Medium |
|  | Tissue: uterus, pre-menopause; Cell type: glandular cells; Level: None; Expression type: APE; Reliability: Medium |
|  | Tissue: vagina; Cell type: squamous epithelial cells; Level: None; Expression type: APE; Reliability: Medium |
|  | Tissue: vulva/anal skin; Cell type: epidermal cells; Level: None; Expression type: APE; Reliability: Medium |
|  | Tissue: cervix |
|  | uterine; Cell type: squamous epithelial cells; Level: None; Expression type: APE; Reliability: Medium |
|  | uterine; Cell type: glandular cells; Level: None; Expression type: APE; Reliability: Medium |

---
